# Borate Transporters and SLC4 Bicarbonate Transporters Share Key Functional Properties

**DOI:** 10.3390/membranes13020235

**Published:** 2023-02-15

**Authors:** Jean L. Beltran, Lila G. McGrath, Sophia Caruso, Richara K. Bain, Claire E. Hendrix, Hana Kamran, Hartlee G. Johnston, Rebecca M. Collings, Menkara-Chinua N. Henry, Tsega-Ab L. Abera, Valeria A. Donoso, Erin C. Carriker, Bryan H. Thurtle-Schmidt

**Affiliations:** Department of Biology, Davidson College, Davidson, NC 28035, USA

**Keywords:** membrane transporters, protein–lipid interactions, Bor1, SLC4A1, yeast

## Abstract

Borate transporters are membrane transport proteins that regulate intracellular borate levels. In plants, borate is a micronutrient essential for growth but is toxic in excess, while in yeast, borate is unnecessary for growth and borate export confers tolerance. Borate transporters share structural homology with human bicarbonate transporters in the SLC4 family despite low sequence identity and differences in transported solutes. Here, we characterize the *S. cerevisiae* borate transporter Bor1p and examine whether key biochemical features of SLC4 transporters extend to borate transporters. We show that borate transporters and SLC4 transporters share multiple properties, including lipid-promoted dimerization, sensitivity to stilbene disulfonate-derived inhibitors, and a requirement for an acidic residue at the solute binding site. We also identify several amino acids critical for Bor1p function and show that disease-causing mutations in human SLC4A1 will eliminate in vivo function when their homologous mutations are introduced in Bor1p. Our data help elucidate mechanistic features of Bor1p and reveal significant functional properties shared between borate transporters and SLC4 transporters.

## 1. Introduction

Bicarbonate transporters in the solute carrier (SLC) 4 family include electroneutral anion exchangers and sodium-coupled cotransporters. The archetypal SLC4 anion exchanger is SLC4A1, also known as Band 3 or Anion Exchanger 1 (AE1). SLC4A1 is the most abundant membrane protein in human red blood cells [1], and reversibly exchanges bicarbonate and chloride ions. Anion exchange can be blocked by stilbene disulfonate-derived inhibitors, and diseases such as renal tubular acidosis, hereditary spherocytosis, and hereditary stomatocytosis can be caused by mutations in the membrane transport domain of SLC4A1 [2]. Despite low sequence identity, SLC4 transporters share structural homology with borate efflux transporters [3,4], which were first discovered in plants [5,6]. Borate is an essential plant micronutrient that enters from the soil and participates in the formation of borate esters that contribute to plant cell wall stability [7,8]. However, excess borate levels are toxic to plants, and thus the regulation of borate by transporters is important for plant viability. Borate levels are regulated partly by BOR1, a borate exporter that promotes borate transport from roots to shoots and leaves by xylem loading [6]. BOR1 is active in *A. thaliana* under limiting borate conditions, but is degraded under high borate concentrations to avoid the accumulation of toxic borate levels in plant shoots [9]. In *S. cerevisiae*, borate is not required for growth, and the function of its borate transporter Bor1p (ScBor1p) is important for mediating resistance to toxic levels of borate [10]. Structures of borate transporters BOR1 from *A. thaliana* [3], Bor1p from *S. mikatae* [4], and BOR3 from *O. sativa* [11], along with structures of SLC4A1 [12,13,14,15], SLC4A4 [16], and SLC4A8 [17], all share the same homodimeric assembly, in which centralized Gate domains mediate dimerization and outer Core domains contain the solute binding pocket between the transmembrane helices TM3 and TM10. Several of these structures occupy different states in the transport cycle, and structural comparisons show that the Gate domains remain relatively rigid throughout the transport cycle while the Core domains are mobile and slide to generate alternating access for bound solutes; these observations are most consistent with an elevator mechanism of transport [3,15,18]. The same architecture of homodimers, composed of Gate and Core domains, has also been seen in the SLC23 orthologs UraA [19,20] and UapA [21], as well as SLC26 proteins like prestin [22] and orthologs BicA [23] and SLC26Dg [24], showing that the SLC4, SLC23, and SLC26 families all share a conserved fold and that insights gained from studying one family may extend to a diverse set of membrane transport proteins.

Despite the structural similarities between borate transporters and SLC4 transporters, one striking difference is that they differ in solute; all but one of the ten human SLC4 transporters transport bicarbonate, while borate transporters instead transport borate. Thus, it remains an open question as to what other biophysical characteristics and functional similarities might extend from SLC4 transporters to borate transporters and vice versa. In particular, the role lipids play in mediating multimeric assembly, whether borate transporters display sensitivity to stilbene disulfonate-derived inhibitors, and structural and mechanistic similarities between amino acids at or near the solute binding site all remain outstanding questions. Because *S. cerevisiae* Bor1p can be overexpressed and purified and has a readily observable phenotype [25], we characterized Bor1p in more detail to learn if its similarities to human SLC4 transporters extend beyond sequence and structural homology.

## 2. Materials and Methods

### 2.1. Protein Overexpression and Purification

ScBor1p was overexpressed and purified as described previously [25]. Briefly, protein constructs bearing C-terminal deca-histidine tags in pRS423-derived plasmids were expressed under galactose-inducible control in *S. cerevisiae* strain BTSY1 (MATα his3::GAL1-GAL4 pep4 prb1-1122) [26]. Cells were lysed by bead beating, and membranes were harvested by differential centrifugation. Following protein solubilization in 1% (*w*/*v*) n-Dodecyl-beta-d-Maltopyranoside (DDM), protein was purified by performing nickel affinity chromatography followed by size exclusion chromatography. Protein was injected onto a Superdex 200 Increase 10/300 GL column (Cytiva) equilibrated in 20 mM Mes pH 6.5, 100 mM NaCl, 2% glycerol, and 0.03% DDM. To compare size exclusion chromatograms between wild-type and mutant ScBor1p, preparations of 1 L of cell culture were grown and harvested under identical conditions.

### 2.2. Proteoliposome Reconstitution and Crosslinking Assays

Purified ScBor1p was reconstituted into lipids by adapting a protocol using preformed vesicles permeabilized with Triton X-100 (EMD Millipore, Billerica, MA, USA) [27]. 1-palmitoyl-2-oleoyl-glycero-3-phosphocholine (PC) and 1-palmitoyl-2-oleoyl-sn-glycero-3-phospho-(1′-rac-glycerol) (PG) were each acquired suspended in chloroform (Avanti Polar Lipids, Alabaster, AL, USA). Lipid solutions were dried to remove chloroform first under nitrogen gas stream and then under vacuum. The dried lipid film was rehydrated at 10 mg/mL in 20 mM Mes (pH 6.5), 100 mM NaCl, and 2% glycerol by incubating at 37 °C for 30 min and resuspending through pipetting. To form unilamellar vesicles, the lipid suspension was subjected to 4 cycles of freeze–thaw, with sonication in a room-temperature water bath for 5 min between freeze–thaw cycles. The lipids were then extruded through a 100 nm filter with 9 passages using a Mini Extruder apparatus (Avanti Polar Lipids, Alabaster, AL, USA). To prepare proteoliposomes, the liposomes were destabilized by adding 0.6% (*v*/*v*) Triton X-100 and were incubated at 4 °C overnight under gentle nutation. Purified ScBor1p was added to the destabilized liposomes at a 1:20 protein:lipid (*w*:*w*) ratio and incubated for 1 h at 4 °C. To extract detergent, SM2 Bio-beads (Bio-Rad, Hercules, CA, USA) were added sequentially in 2 steps. First, 50 mg SM2 Bio-beads were added and incubated for 1 h at 4 °C, followed by the addition of another 50 mg of SM2 Bio-beads and another incubation for 1 h at 4 °C. The glutaraldehyde cross-linking assay was performed as described previously [25], with cross-linking performed for 5 min at room temperature with 0.15% glutaraldehyde (Electron Microscopy Sciences, Hatfield, PA, USA) in the presence or absence of a 5 min pre-treatment of 2% (*w*/*v*) sodium dodecyl sulfate (SDS) detergent (Sigma-Aldrich, St. Louis, MO, USA).

### 2.3. Genetic Plating Assay

The genetic plating assay was performed as described previously [26]. A yeast strain (BTSY2) with a knocked out BOR1 (MATα his3::GAL1-GAL4 pep4 prb1-1122 bor1::KanMX) was transformed with pRS423-derived plasmids that are under inducible control of the GAL1 promoter and possess a C-terminal deca-histidine tag. Plasmids differ only by the transporter they encode or the specified mutation they bear. Overnight cultures were grown in media consisting of yeast nitrogen base (YNB + nitrogen), complete supplement mixture lacking histidine, and were supplemented with adenine (CSM-His w/Ade40), with 2% raffinose for a sugar source. Cells were diluted to an OD600 of 0.5 and then serial fivefold dilutions were prepared and added to plates containing CSM-His w/Ade40, 2% raffinose, 0.05% galactose to induce expression, and either 0 mM, 10 mM, or 20 mM boric acid to challenge yeast growth. 10 μL of each dilution was plated, and plates were stored at 30 °C for 5 days until imaged. Images presented here are representative plates from at least three biologically independent replicates. For the SITS and H_2_DIDS experiments, the protocol was identical. However, per L of media we used 1 g monosodium glutamate instead of 5 g ammonium sulfate for a nitrogen source. Additionally, inhibitors were prepared at 50 mM in dimethyl sulfoxide (DMSO), which permitted adding up to 300 μM inhibitors with only 0.6% DMSO present. Therefore, 0.6% DMSO was used in all other lesser inhibitor concentrations and control conditions.

### 2.4. Borate Quantification Assay

The spectrophotometric assay to quantify borate from cell lysates was adapted from a previously published protocol [28]. Yeast colonies were used to inoculate overnight cultures, which were then seeded to an OD600 of 0.25 the following morning and allowed to grow for 7 h until the OD600 was around 0.9. Protein expression was induced for 16 h by the addition of 2% galactose, after which cultures had 1 mM boric acid added for 90 min. Cells were pelleted and washed with water before being pelleted a second time, resuspended in 350 μL water, and lysed by incubating at 98 °C for 30 min. After a 5 min spin at 16,100× *g*, 300 μL of supernatant was prepared for curcumin addition per the previously reported protocol, with the data collected using a quartz cuvette that was washed twice with 91% isopropyl alcohol in between measurements. Standard curves were generated from standards containing 0, 0.625, 1.25, 2.5, and 5.0 mg/L of borate. To avoid the risk of borate entering the experiment via borosilicate glassware, no solutions in these experiments touched glass. 95% confidence intervals were generated for *n* = 7 biologically independent experiments.

### 2.5. Western Blot

Protein overexpression protocols were adapted from those described above, but with the following distinctions: 50 mL yeast cultures were grown using strain BTSY1, and after 16 h induction by 2% galactose the cells were lysed by passing once through an Emulsiflex-B15 homogenizer (Avestin, Ottawa, ON, Canada) at 22,500 psi. Membranes were collected by differential centrifugation and resuspended in 20 mM Tris pH 7.4, 1 mM EDTA, 1 mM PMSF, 10% glycerol, and 300 mM NaCl. For Western blot analysis, 10 μg total protein was loaded per lane, and proteins were transferred to a PVDF membrane in a wet tank. The anti-His_6_, rabbit polyclonal primary antibody (Fisher, Waltham, MA, USA, Cat# PIPA1983B) was incubated at 4 °C overnight, and the HRP-conjugated goat anti-rabbit secondary antibody (Invitrogen, Waltham, MA, USA, Cat# 32460) was incubated at room temperature for one hour before exposure using chemiluminescent horseradish peroxidase substrate to image. Total protein loading controls were imaged using Bio-Rad stain-free gels.

## 3. Results and Discussion

### 3.1. A Dispensable N-Terminal Tail and a Conserved Functional Membrane Transport Fold

**Figure 1:**
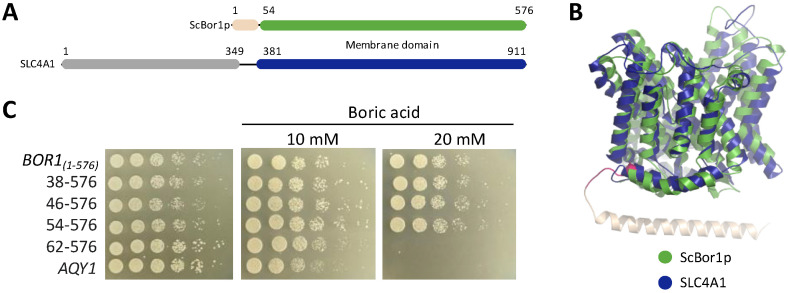
An N-terminal region is dispensable for ScBor1p function. (**A**) Schematic showing the domain arrangements of ScBor1p and human SLC4A1. The membrane domains are in green and blue, respectively. SLC4A1 cytosolic ankyrin-binding domain is in gray. (**B**) Superposition of a cryoEM structure of the membrane domain (blue) of human SLC4A1 (PDB ID: 8CT3) [14] and the AlphaFold model of ScBor1p (green) rendered in PyMOL with RMSD = 2.993 Å. The dispensable region of the N-terminal tail is tan; the region that results in no function when deleted is pink and marks the beginning of helix H1. (**C**) Plasmids encoding the specified ScBor1p truncated construct or negative control *AQY1* were transformed into *bor1* deletion cells and the ability of each to rescue growth was tested by plating fivefold serial dilutions onto plates containing CSM-His selective media and the indicated boric acid concentrations. Plates were incubated at 30 °C and imaged after 5 days.

All ten transporters in the human SLC4 family are composed of an N-terminal cytoplasmic domain linked to a C-terminal membrane transport domain. A sequence alignment of ScBor1p with human SLC4A1 shows just over 24% sequence identity between their transporter domains (Figure S1). Fungal borate transporters lack the cytoplasmic domain of SLC4 transporters, and ScBor1p instead has a ~50 amino acid N-terminal tail that differentiates it from plant borate transporters, as well as human SLC4 orthologs (Figure 1A). The structural model of ScBor1p from the AlphaFold server predicts this N-terminal region to be composed of a long alpha helix running parallel to the membrane and preceding helix H1 (Figure 1B) [29], but any functional significance of this region is unknown. To test whether this N-terminal region is important for function, we generated N-terminally truncated constructs and tested them for phenotypes in a genetic plating assay. In this experiment and those to follow, plates containing no boric acid are controls that are expected to show equivalent growth for all samples. Only cells that express a functional borate transporter can grow on the plates with 20 mM boric acid, while plates containing 10 mM boric acid can identify more subtle phenotypic changes. We used the nonfunctional yeast aquaporin AQY1 as a negative control [30]. Our results show that the N-terminal region of ScBor1p is not essential through the first 53 residues but that truncating the first 61 amino acids results in a total loss of ability to grow on plates containing 20 mM boric acid (Figure 1C). A previous study in the membrane domain of human SLC4A1 showed that deleting amino acids 381–385 results in lost transport activity [31]. Residues 381–385 in human SLC4A1 and residues 54–61 in ScBor1p overlap by one amino acid in a multiple sequence alignment (Figure S1), and both regions encapsulate the beginning of helix H1 (Figure 1B). Our data demonstrate that the first 53 amino acids of ScBor1p are dispensable for in vivo function, and that borate transporters and SLC4 transporters share a conserved functional core in which the beginning of helix H1 is important for function.

### 3.2. Lipids Promote ScBor1p Dimerization

**Figure 2:**
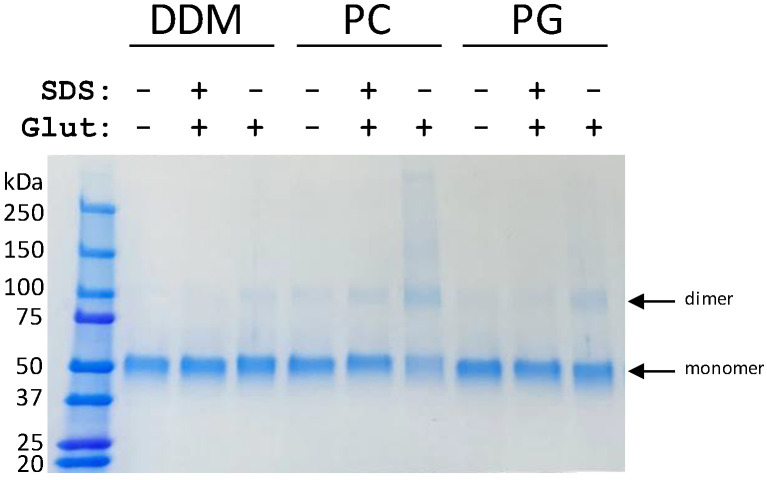
Cross-linking shows lipid-promoted dimerization of ScBor1p. It is indicated above each lane whether the sample is in DDM or reconstituted with PC or PG lipid. “SDS” indicates a 5-min pre-treatment of 2% sodium dodecyl sulfate before the addition of 0.15% glutaraldehyde for 5 min.

ScBor1p could be readily purified as described previously [25]. When solubilized and purified in DDM, our group and others have shown that ScBor1p is almost entirely monomeric [25,32]. However, previous studies have determined experimental structures of *A. thaliana* Bor1 and the yeast *S. mikatae* Bor1p (SmBor1p) in dimeric assemblies [3,4], and SmBor1p has 89% sequence identity to ScBor1p (Figure S1). To better understand the physical basis of ScBor1p monomer-dimer equilibrium, we prepared proteoliposomes and performed a glutaraldehyde covalent cross-linking assay that we have previously used to assess dimerization in detergent [25]. We tested phosphatidylcholine (PC) because it is the most abundant glycerophospholipid in *S. cerevisiae* [33], and we used phosphatidylglycerol (PG) as a control because a previous study showed that it promoted ScBor1p dimerization, despite its typical absence in yeast membranes [32]. The PC and PG preparations each contain identical mixed acyl fatty acid chains of 16:0 and 18:1, so the lipids differ only in their head group identity. Our results show that PC partially shifts the sample towards dimerization, while PG results in a more modest shift than PC (Figure 2). Controls in which SDS was added prior to the addition of glutaraldehyde demonstrate that the dimerization we observe is minimal in denaturing conditions.

A previous study on ScBor1p showed that PS and PE each shift ScBor1p to just under 50% dimer, while PC did not support dimerization [32]. There are several notable differences between the two approaches. In the prior study, detergent-solubilized protein had lipid added to it and then was subjected to mass spectrometry. In our study, protein was reconstituted into proteoliposomes, detergent was removed, and dimerization was assessed by a glutaraldehyde cross-linking assay [20,25]. The glutaraldehyde cross-linking assay depends on the presence of primary amino groups from lysines in proximity to one another. Phosphatidylserine (PS) and phosphatidylethanolamine (PE) each possess a primary amino group and are therefore incompatible with a glutaraldehyde-based crosslinking assay. There are several possible interpretations of our observed shift from PC compared to the absence of dimer observed from PC in the former study. One interpretation is that proteoliposomes are closer to physiological conditions than those performed in the other study, which could explain the greater dimerization seen for PC in our experiment. Likewise, perhaps ScBor1p assembly into PS or PE proteoliposomes would shift the equilibria further towards dimer, but we cannot assess that due to PS and PE’s incompatibility with glutaraldehyde. A second possibility is that no one lipid drives the equilibrium towards complete dimerization, but that a more complex lipid mixture, like what occurs in vivo, could. Further studies will be required to understand the relationships between lipid binding and multimerization. Our data and the prior study are in agreement that multiple lipids can promote dimerization, though not to more than half of the sample in tested conditions. Interestingly, experimental structures of human SLC4A1 show lipids bind in the crevice in between Gate domains at the dimerization interface [13,14]. Additionally, the structurally similar SLC23 homolog UapA has been shown to display lipid-mediated dimerization [34], and the human SLC26 protein prestin has been shown to have cholesterol bridge contacts between its dimer interface [22]. Likewise, the fumarate transporter SLC26Dg purifies as a monomer in DDM but is observed to show dimers in lipids [24,35], just as we observe for ScBor1p. Lipid-mediated multimeric assembly between Gate domains may therefore be broadly conserved among the SLC4, SLC23, and SLC26 families.

### 3.3. Sensitivity to Derivatives of Stilbene Disulfonate

**Figure 3:**
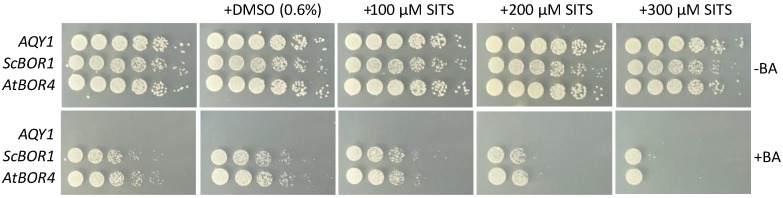
Borate transporters are sensitive to SITS. Plasmids encoding the specified gene were tested against increasing concentrations of SITS in 0.6% dimethyl sulfoxide (DMSO) in the presence or absence of 20 mM boric acid and plated on CSM-His selective media. Plates were incubated at 30 °C and imaged after 5 days.

One hallmark feature of human SLC4 transporters is the inhibition of their anion transport by stilbene disulfonate-derived inhibitors [36,37]. If ScBor1p is a strong model for studying SLC4 transporters, we predict that borate transporters would be inhibited by stilbene disulfonate-derived inhibitors such as 4-acetamido-4′-isothiocyanatostilbene-2,2′-disulfonic acid (SITS). To determine whether borate transporters can be inhibited by SITS, we tested the sensitivity of ScBor1p and the *A. thaliana* Bor4 transporter (AtBOR4) to SITS through genetic plating assays. We chose AtBOR4 both because the sensitivity of plant borate transporters to SITS is unknown and because a prior study showed that among all seven *A. thaliana* borate transporters, BOR4 shows the strongest growth phenotype against boric acid in *bor1* deletion cells [26]. Our results show that ScBor1p and AtBOR4 are each inhibited by SITS (Figure 3). Importantly, negative controls show that the highest SITS concentration tested has no deleterious effect on growth in plates lacking boric acid, demonstrating that SITS toxicity is specific to the cellular context in which borate transport is necessary for survival. A plating assay with 4,4′-diisothiocyanatodihydrostilbene-2,2′-disulfonic acid (H_2_DIDS) shows borate transporter sensitivity, though H_2_DIDS also shows some toxicity in non-borate testing conditions. Therefore, the effect of H_2_DIDS on ScBor1p is less clear (Figure S2). A previous study showed that ScBor1p could bind to a resin conjugated to SITS [38]; here we show in vivo evidence demonstrating that both ScBor1p and AtBOR4 are inhibited by SITS. Borate transporter similarities with SLC4 transporters thus include sensitivity to the same small molecule inhibitors.

### 3.4. Identifying Functional Amino Acids at the Solute Binding Site

**Figure 4:**
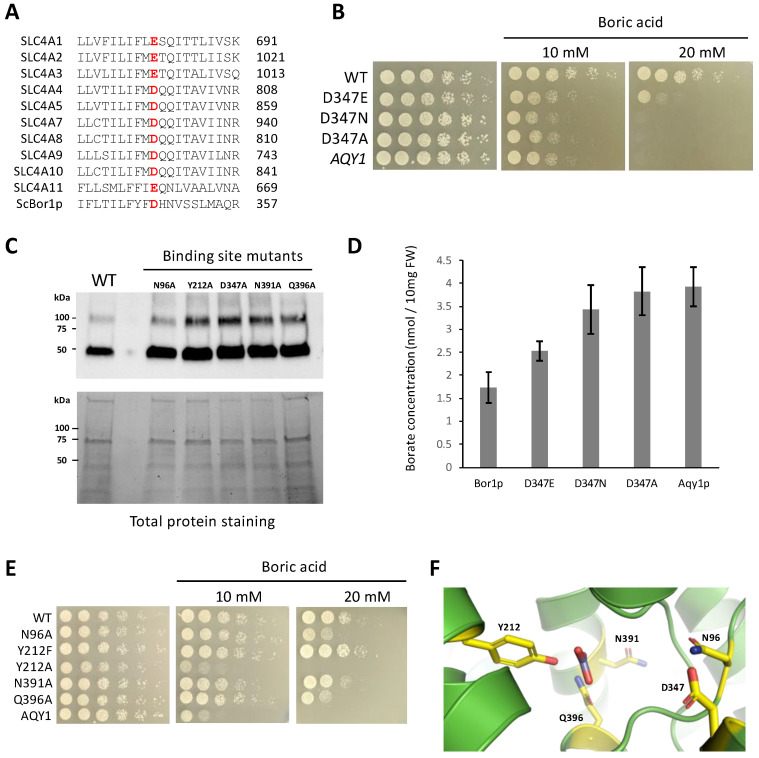
Identification of amino acids important for Bor1p function. (**A**) Multiple sequence alignment of a region of TM8 for all 10 human SLC4 transporters and ScBor1p. (**B**,**E**) Plasmids encoding the specified ScBor1p mutant were tested against the indicated boric acid concentrations. Plates were incubated at 30 °C and imaged after 5 days. (**C**) Western immunoblotting analysis of His-tagged ScBor1p. (**Top**) Samples were analyzed with an anti-His6-tag antibody. (**Bottom**) Total protein stain-free gel imaging of 12μg protein per lane served as loading controls. (**D**) Quantification of borate efflux activity in yeast cells expressing each indicated protein. Intracellular borate contents are reported as nmol per 10 mg fresh weight (FW) of yeast cells. Error bars represent 95% confidence intervals for *n* = 7 biologically independent experiments. (**F**) View of the solute-binding site of the ScBor1p AlphaFold model with tested amino acids in yellow. Bicarbonate is displayed from a superposed human SLC4A1 structure (RMSD = 3.149 Å).

Previous studies in SLC4A1 show that the solute binding site contains a glutamate, E681 in human numbering, that is essential for function [39,40,41]. An acidic residue in this position is perfectly conserved in the SLC4 family (Figure 4A), and previous studies have suggested that the difference between the presence of a glutamate or aspartate in this position could influence whether the transporter is an anion exchanger or a sodium co-transporter, respectively [16]. In borate transporters, this position is an invariant aspartate. Previous work has shown that alanine substitutions at position D347 in ScBor1p and the homologous D311 in AtBor1 each abolish function [3,11]. To test how more conservative changes to D347 might impact function, we assayed the effect of D347E and D347N in genetic plating assays. Interestingly, we show that the D347E mutation results in a hypomorphic phenotype, while a mutation as conservative as D347N results in no growth (Figure 4B). Western blot analysis shows that the least conservative mutation, D347A, shows robust expression like the wild-type protein, and therefore a decrease in expression cannot explain the phenotype (Figure 4C). Because we previously reported that AtBOR1 supports no growth in yeast plated on media containing 20 mM boric acid but can nevertheless display borate efflux activity through assaying borate quantities in cell lysates [26], we tested whether these amino acid changes to D347 directly affected borate efflux activity by using a spectrophotometric assay for quantifying borate [28]. Our results show that the D347E mutation results in decreased but significant borate transport relative to negative controls, while the D347N mutation abolishes borate transport to levels indistinguishable from negative controls (Figure 4D). Taken together, the multiple sequence alignment, genetic data, and borate quantification data suggest a requirement for an acidic residue at this location in borate transporters and SLC4 transporters alike. That the most conservative mutation tested, D347N, results in a total loss of function suggests that D347 may be involved as a proton donor/acceptor. 

Having examined D347, the amino acid most established to be critical for transport, we next wanted to identify other proximal amino acids that might be important for the solute binding and transport mechanism of ScBor1p. We performed alanine scanning mutagenesis on amino acids that are conserved in borate transporters (Figure S1) and adjacent to the cavity predicted to bind to borate and any co-transported ions. We selected hydrophilic amino acids with the assumption that they were most likely to interact directly with a polar solute. As controls we included N391A and Q396A, which were previously shown to result in reduced rescue on plates with boric acid [3]. Here we identify N96A as displaying a hypomorphic phenotype. Additionally, the Y212A substitution eliminates growth entirely, while the more conservative Y212F mutation has no effect (Figure 4E). Western blot analysis of membrane fractions shows that these mutants have robust expression and therefore their phenotypes cannot be explained by reduced protein expression (Figure 4C). Our data here identify a constellation of amino acids important for ScBor1p function. Might any of these residues interact with solute? There is no structure of a borate transporter bound to its solute, so we performed a superposition of a structure of bicarbonate-bound human SCL4A1 [42] with the AlphaFold ScBor1p model (RMSD = 3.149 Å) (Figure 4F) [29]. As suspected, the bicarbonate places in the cavity formed where TM3 and TM10 meet. Among amino acids identified in this study, Q396 and Y212 are closest to where bicarbonate superposes. In human SLC4A1, R730 is homologous with Q396 and is observed to interact directly with bicarbonate [42]. Interestingly, the carboxylate group of D347 is located 8.2 Å away from the superposed bicarbonate carbon. There is room to accommodate a sodium ion in the space between, but a previous study suggests that sodium is not coupled with borate transport in ScBor1p [43]. That same study suggested that protons are the ion whose favorable transport is coupled with the pumping of borate against its gradient. Our observation of no function for the D347N suggests it is possible that D347 is involved in proton binding.

### 3.5. Disease-Causing Mutations from SLC4A1 Also Eliminate ScBor1p Function

**Figure 5:**
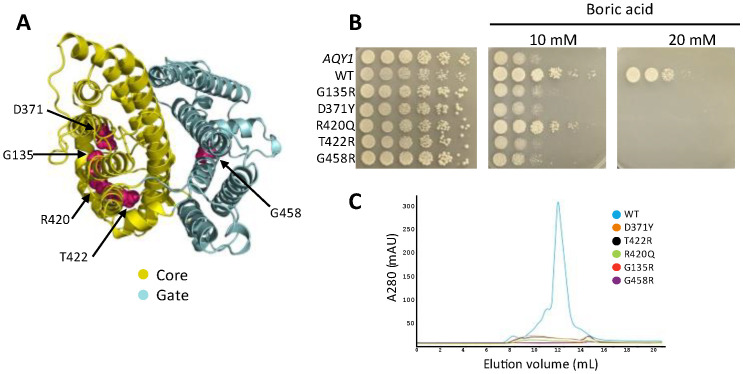
Impacts of disease-causing mutations in human SLC4A1 transfer to ScBor1p. (**A**) AlphaFold model of ScBor1p with homologous locations of disease-causing mutations in SLC4A1 in pink. (**B**) Plasmids encoding the specified ScBor1p mutant were tested against the indicated boric acid concentrations. Plates were incubated at 30 °C and imaged after 5 days. (**C**) Superposed chromatograms from size-exclusion chromatography performed for wild-type and mutant ScBor1p.

Mutations in human SLC4A1 are known to lead to several genetic disorders, including hereditary spherocytosis and hereditary stomatocytosis [2]. Prior work has shown that two disease-causing mutations in human SLC4A1, S762R and G796R, result in loss of borate transport when the homologous mutations, S466R and A500R, are introduced in AtBOR1 [11]. To test whether these same homologous disease-causing mutations impact ScBor1p and whether additional disease-causing mutations have deleterious effects on ScBor1p function, we tested the above two mutations, as well as an additional three amino acid substitutions that are linked to disease in humans and are conserved in ScBor1p (Figure 5A and Figure S1). The aforementioned S762R and G796R are linked to hereditary stomatocytosis [44,45], and their homologous mutations in ScBor1p are T422R and G458R, respectively. Additionally, we identified three mutations in SLC4A1 that are linked to hereditary spherocytosis—G455R, D705Y, and R760Q [46,47,48]—which have homologous mutations in ScBor1p—G135R, D371Y, and R420Q, respectively (Figure 5A and Figure S1). We tested all five mutations in a genetic plating assay and show that all five mutants fail to rescue growth on plates containing 20 mM boric acid (Figure 5B). To see what effect these mutations have on protein expression, we subjected each mutant ScBor1p to identical protein expression, solubilization, and purification protocols [25], and then compared their size-exclusion chromatograms. The data show a severe decrease in protein for all mutants (Figure 5C). Interestingly, the R420Q mutant promotes growth on the 10 mM plates but does not express better than the other four mutant proteins. A previous study of the SLC4A1 mutant R760Q (homologous with R420Q) showed an absence of R760Q mutant protein detected in the red blood cell membranes of a patient bearing the R760Q mutation in one of their alleles [48]. These data suggest that the five mutations have impacts on the folding, stability, or trafficking of ScBor1p that could lead to lost in vivo function, though it is possible the mutant proteins express at meaningful levels in the cell but cannot be readily solubilized and purified in DDM detergent. Nonetheless, mutations equivalent to disease-causing mutations in SLC4A1 have significant deleterious effects on the in vivo function of ScBor1p.

## 4. Conclusions

When borate transporters were first discovered, the sequence similarity between them and human SLC4 transporters was noted immediately [6]. More than a decade later, experimentally determined structures began to emerge for SLC4A1 [12,13,14,15], SLC4A4 [16], SLC4A8 [17], AtBOR1 [3], OsBOR3 [11], and SmBor1p [4]. Although an obvious difference exists between the solutes of these transporters—borate transporters transport borate while all but one of the ten SLC4 transporters transport bicarbonate—the structural similarities are striking, as all of them share the same homodimeric assembly composed of Gate and Core domains. Our work here shows that the similarities extend beyond their sequence and structure to include lipid-promoted dimerization, sensitivity to stilbene disulfonate-derived inhibitors, a requirement for an acidic amino acid at the solute binding site, and the conservation of deleterious impact from disease-causing mutations. 

Studies of the one SLC4 transporter that does not transport bicarbonate, SLC4A11, reveal additional connections between the SLC4 family and borate transport. When SLC4A11 was first characterized it was initially proposed to transport borate [49], but this claim proved controversial and evidence has since emerged that human SLC4A11 cannot transport borate but rather is likely to transport H^+^/OH^−^ [26,50,51,52]. A recent report, however, shows that seawater fish use their SLC4A11 ortholog to excrete boric acid in the kidneys, suggesting that, during the history of vertebrate evolution, either mammalian SLC4A11 lost boric acid transport activity or saltwater fish acquired boric acid transport activity [53]. Further studies are likely to establish more evolutionary, structural, and mechanistic connections between borate transporters and the SLC4 family.

## Data Availability

The data that support the findings of this study are available on request from the corresponding author, B.H.T.-S.

## Supplementary Materials

The following supporting information can be downloaded at: https://www.mdpi.com/article/10.3390/membranes13020235/s1, Figure S1: Alignment of borate transporters with human SLC4A1; Figure S2: Sensitivity to H_2_DIDS.
