# Supplementary material for: Borate Transporters and SLC4 Bicarbonate Transporters Share Key Functional Properties
Source: Membranes (Basel). 2023 Feb 15;13(2):235. doi: 10.3390/membranes13020235 (PMC9959716; doi:10.3390/membranes13020235)
Supplement: Supplementary file 1 [file membranes-13-00235-s001.zip › membranes-2212436-supplementary.pdf]

A

```
                                                                                  Helix H1
HsSLC4A1   ------------------------------------------------------------GLVRDIRRRYPYYLS   395
AtBOR1     -------------------------------------------------MEETFV--PFEGIKNDLKGRLMCYKQ   24
SmBor1p    MLKDSTGVTISRGFTASRECTQPSEETNDEPNTESSVVDSRNDEESHEKLSRRRLPKLGMGIWLDLKDRIPYYKS   75
ScBor1p    MSNESTRVTVSRGCTASDECAQALERTNDELDRESSVSESRSDEESHEKLSRRRFPTLGIGIWLDLKDRIPYYKS   75

                                  TM1                              TM2
HsSLC4A1   DITDAFSP--QVLAAVIFIYFAALSPAITFGGLLGEKTRNQMGVSELLISTAVQGILFALLGAQPLLVVGFSGPL   468
AtBOR1     DWTGGFKAGFRILAPTTYIFFASAIPVISFGEQLERSTDGVLTAVQTLASTAICGMIHSIIGGQPLLILGVAEPT   99
SmBor1p    DWVDAFNY--RVIPSTVDTYFNNLLPAIAFAQDMFDRTDNSYGVNEVLLSSAMAGIVFGVLAGQPLCIVGVTGPI   148
ScBor1p    DWVDAFNY--RVIPSIVDTYFNNLLPAIAFAQDMFDRTDNSYGVNEVLLSSAMAGIVFGVLGGQPLCIVGVTGPI   148
                                N96                                        G135
       TM3                         TM4                                         TM5
HsSLC4A1   LVFEEAFFSFCETNG----LEYIVGRVWIGFWLILLVVLVVAFEGSFLVRFISRYTQEIFSFLISLIFIYETFSK   539
AtBOR1     VIMYTFMFNFAKARPELGRDLFLAWSGWVCVWTALMLFVLAICGACSIINRFTRVAGELFGLLIAMLFMQQAIKG   174
SmBor1p    SIFNYTVYEIIKPLN----TSYFGFMFWICLWSMIFHLLLAFTNVVCLLQYVTTFPCDIFGLFINVVYIQKGIQI   219
ScBor1p    SIFNYTVYEIIKPLN----TSYFGFMFWICMWSMIFHLVLAFTNAVCLLQYVTTFPCDIFGLFINVVYIQKGIQI   219
                                                                                Y212
                                                    TM6
HsSLC4A1   LIKIFQDHPLQKTYNYNVLMVPKPQGPLPNTALLSLVLMAGTFFFAMMLRKFKNSSYFPGKLRRVIGDFGVPISI   614
AtBOR1     LVDEFRIPERENQK-LKEF---LPSWRF-ANGMFALVLSFGLLLTGLRSRKARSWRYGTGWLRSLIADYGVPLMV   244
SmBor1p    LTRQFHNTSGEKSV---------------QDGFASVVVALVMTAFGLFFKSFHHYPLFTHKIRTFISDYSTALSV   279
ScBor1p    LTRQFSAKSGEKSV---------------QDGFASVVVALVMTAFGLFFKLFHYYPLFSHRIRTFISDYSTALSV   279

     TM7                                                                       TM8
HsSLC4A1   LIMVLVDFFIQ---DTYTQKLSVPDGFKVSNSSARGWVIHPLGL------RSEFPIWMMFASALPALLVFILIFL   680
AtBOR1     LVWTGVSYI---------PAGDVPKGIPRRLFSPNPWSPGAYGNWTVVKEMLDVPIVYIIGAFIPASMIAVLYYF   315
SmBor1p    LFWSSFTHFGGYLNDVKFKKLPITK-----SFFPTSKFNRPQNTWLAYE---PIPVKDVFIALPFGIILTILFYF   346
ScBor1p    LFWSSFTHFGGYLHDVKFKKLPITK-----AFFPTSKVNRPQNTWLAYE---PIPVKDVFIALPFGIFLTILFYF   346

                                  TM9                TM10
HsSLC4A1   ESQITTLIVSKPERKMVKGSGFHLDLLLVVGMGGVAALFGMPWLSATTVRSVTHAN-------------------   736
AtBOR1     DHSVASQLAQQKEFNLRKPSSYHYDLLLLGFLTLMCGLLGVPPSNGVIPQSPMHTKSLATLKYQLLRNRLVATAR   385
SmBor1p    DHNVSSLMAQRHQYKLRKPSSFHYDFALLGLTTCISGVLGIPAPNGLIPQAPLHTETLLVRDS------------   409
ScBor1p    DHNVSSLMAQRHQYKLKKPSSFHYDFALLGLTTCISGVLGIPAPNGLIPQAPLHTETLLVRDS------------   409
         D347                     D371                N391  Q396A

HsSLC4A1   ---------------------------------------ALTVMGKASTPG-----------AAAQIQEV   756
AtBOR1     RSIKTNASLGQLYDNMQEAYHHMQTPLVYQQPQGLKELKESTIQATTFTGNLNAPVDETLFDIEKEIDDLLPVEV   460
SmBor1p    --------------------------------------------------------------------NQNVVRC   416
ScBor1p    --------------------------------------------------------------------NQKVISC   416

               TM11                         TM12
HsSLC4A1   KEQRISGLLVAVLVGLSIL--MEPILSRIPLAVLFGIFLYMGVTSLSGIQLFDRILLLFKPPKYHPDVP------   823
AtBOR1     KEQRVSNLLQSTMVGGCVA--AMPILKMIPTSVLWGYFAFMAIESLPGNQFWERILLLFTAPSRRFKVLEDYHAT   533
SmBor1p    VEQRLTNTFQGLMILGTMTRPLLVCLGEIPQAVLSGLFFIMGINGLMTNVIIHRIVFLFSDPKRRDNNSPL----   487
ScBor1p    VEQRFTNTFQGLMILGTMTRPLLVCLGEIPQAVLSGLFFIMGINGLMTNSIIQRLVFLFSDPNRRDNTSPL----   487
          R420 T422                           G458
                      TM13                       TM14
HsSLC4A1   YVKRVKTWRMHL----FTGIQIICLAVLWVVKSTPASLALPFVLILTVPLRRVLLPLIFRNVELQCLDADDAKAT   894
AtBOR1     FVETVPFKTIAMFTLFQTTYLLICFGLTWIPI---AGVMFPLMIMFLIPVRQYLLPRFFKGAHLQDLDAAEYEEA   605
SmBor1p    --AKISKRSMVIF----LCFSLAGFTGEFAITNTIAAIGFPLVLLLSVIVS-FSFTYIFPSEELKVLDNNVAQRF   555
ScBor1p    --MKVSKKSMLIF----LSFSLTGFAGEFAITNTIAAIGFPLVLLLSVLVS-FSFAYIFPTEELKILDTNVAQKF   555

HsSLC4A1   FDE--------EEG--------------------RDEYDEVAMPV-   911
AtBOR1     PALPFNLAAETEIGSTTSYPGDLEILDEVMTRSRGEFRHTSSPKV   650
SmBor1p    TLKNLLLENI-----------------RDAKFSDRHKF--------   576
ScBor1p    TIKNLLLENI-----------------RDAKFCDKHED--------   576
```

B

	ScBor1p	SmBor1p	AtBOR1	HsSLC4A1
ScBor1p	100.00%	88.89%	27.79%	24.69%
SmBor1p	88.89%	100.00%	27.98%	24.69%
AtBOR1	27.79%	27.98%	100.00%	28.24%
HsSLC4A1	24.69%	24.69%	28.24%	100.00%

Figure S1. Alignment of borate transporters with human SLC4A1. (**A**) Alignment was performed in UniProt using the full-length sequences for ScBor1p, SmBor1p, AtBOR1, and the membrane domain sequence for human SLC4A1. Marked in red are residues investigated in this study, with S. cerevisiae numbering below. Helix H1 and all 14 transmembrane helices are indicated above their respective sequences. In blue are regions which when deleted result in lost function. (**B**) UniProt output for the percent identity matrix for the four sequences.

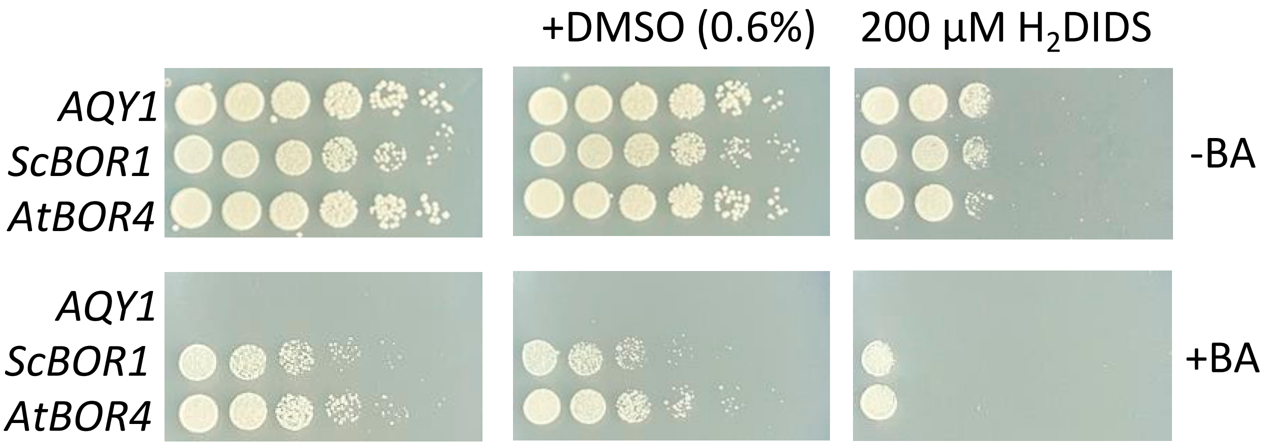

Figure S2. Sensitivity to H_2DIDS. Plasmids encoding the specified gene were tested against H_2DIDS in 0.6% DMSO or just 0.6% DMSO in the presence or absence of 20mM boric acid and plated on CSM-His selective media. Plates were incubated at 30°C and imaged after 5 days.
